# 

**Published:** 1899-03

**Authors:** 


					CONTENTS.
The Surgical Treatment of Myoma
of the Uterus.
By W. Roger
Williams, F.R.C.S.
A Case of Hysterectomy for Fibroid
Tumour of Uterus, with Intra-
Abdominal Treatment of Pedicle.
By W. H. C. Newnham, M.A., M.B.
Cantab.
Symphysiotomy.
By Walter C.
Swayne, M.D. Lona.
A Case of Acute Ulcerative Endo-
carditis.
?/T
M.D.R.U.I., M.R.C.S., L.R.C.P.
IS
Achondroplasia.
By Charles E. S.
Flemming, M.R.C.S., L.R.C.P.
21
The Bacteriology of Some Suppura-
tions Complicating Pulmonary
Disease.
By J. O. Symes, M.D.,
D.P.H.
30
Progress of the Medical Sciences:
Medicine.
Theodore Fisher, M.D.
Surgery.
J. Lacy Firth, M.D.
Ophthalmology.
Cyril H.
Walker, M.B.
49
Reviews of Books
so
Notes on Preparations for the Sick
77
Bristol Medico-Chirurglcal Society
79
The Library of the Bristol Medico-
Chirurgical Society  89
Local Medical Notes
92
Scraps
93

				

